# Family roles in informed consent from the perspective of young Chinese doctors: a questionnaire study

**DOI:** 10.1186/s12910-023-00999-6

**Published:** 2024-01-03

**Authors:** Hanhui Xu, Mengci Yuan

**Affiliations:** School of Medicine, Nankai University, Tianjin, 300071 China

**Keywords:** Informed consent, Family roles, Physician-patient relationship, Chinese young doctors

## Abstract

**Background:**

Based on the principle of informed consent, doctors are required to fully inform patients and respect their medical decisions. In China, however, family members usually play a special role in the patient’s informed consent, which creates a unique “doctor-family-patient” model of the physician-patient relationship. Our study targets young doctors to investigate the ethical dilemmas they may encounter in such a model, as well as their attitudes to the family roles in informed consent.

**Methods:**

A questionnaire was developed including general demographic characteristics, the fulfillment of the obligation to fully inform, who will be informed, and the ethical dilemmas in decision-making. We recruited a total of 421 doctors to complete this questionnaire, of which 368 met the age requirements for this study. Cross tabulation and Pearson’s chi-squared test were used to analyze the differences between types of patients for categorical variables, and a *p*-value < 0.05 was considered statistically significant.

**Results:**

Our data shows that only 20 doctors (5.40%) stated “informing the patient alone is sufficient” when it comes to informing patients of their serious conditions. The rest of the participants would ensure that the family was informed. When facing elderly patients with decision-making capacity, the data was statistically different (3.8%; P < 0.001) The primary reason for ensuring that family members be informed differs among the participants. In addition, when family members asked doctors to conceal the patient’s medical condition for the best interests of patients, 270 doctors (73.4%) would agree and cooperate with the family. A similar proportion (79.6%) would do so when it comes to elderly patients.

**Conclusions:**

(1) Chinese doctors pay extra attention to informing the patient’s family, which may not be in the patient’s best interests. (2) Chinese doctors treat adult (but not elderly) patients and elderly patients differently when it comes to informing family members. (3) When family members request that doctors withhold information from patients “in the best interest of the patient,” the majority choose to comply with the request, although this may cause them distress.

**Supplementary Information:**

The online version contains supplementary material available at 10.1186/s12910-023-00999-6.

## Background

The purpose of informed consent is to protect patients’ autonomy. In the West, under the principle of informed consent, doctors are required to provide patients with adequate information and respect their decisions [1, 2]. The patient’s family members may take part in the treatment process. However, on the doctor’s side, the patient’s autonomy and rights are always the primary consideration. They will not disclose a patient’s condition to family members without the patient’s consent. It is even less likely for doctors to bypass patients and prioritize informing the family members of their condition. Only in special circumstances, such as when a patient loses the ability to make decisions, will the doctors then inform the family members and act on their decisions [3, 4].

In China, however, it is customary for doctors to inform both the patient and their families of the patient’s condition, even if the patient is capable of making decisions. This usually happens when the family members accompany the patient or when the patient’s condition is complex and severe [5, 6]. In some special cases, doctors will even give priority to informing family members of the condition and letting them communicate with the patient [7, 8]. In other cases, family members believe that disclosing the true condition will cause significant psychological harm to the patient, and they may request that doctors cooperate with the family in “deceiving” the patient. Most of the time, Chinese doctors will accede to the family’s request [9–11].

Some scholars claim that family members play such an important role in contemporary Chinese medical practice because of the influence of the traditional Confucian culture [12–14]. Confucian culture tends to view the family as the basic unit of society, as opposed to the current Western society, which views the individual as the basic unit of society. Major decisions regarding personal well-being are often made collectively by family members. This family-oriented ideology has shaped a unique “doctor-family-patient” model of the physician-patient relationship, in which physicians are no longer dealing solely with the patient, but also with the patient’s family. Countries and regions influenced by Confucian cultures, such as Japan, South Korea, and Hong Kong, also have similar situations where family members participate in medical decision-making [15–18]. Additionally, scholars have analyzed this physician-patient relationship model from an economic, medical insurance policy, and educational perspective to explain its realistic basis [5, 19].

Research on this “doctor-family-patient” model of the physician-patient relationship has primarily focused on three areas: (1) investigating the attitudes and reactions of patients and their families toward family involvement in informed consent and medical decision-making; [10, 19, 20] (2) analyzing the underlying reasons for this model; [5, 12, 19, 21] and (3) evaluating the advantages and disadvantages of this model [22–25]. However, existing research lacks empirical studies on the physician population. This appears to neglect the views of physicians on this doctor-patient relationship model and the potential challenges it may pose for them. On the one hand, Chinese physicians are educated and trained to protect the patient’s privacy, fulfill their duty of informed consent, and respect the patient’s autonomy, with such requirements reflected in relevant laws [26, 27]. On the other hand, in China, physicians appear to comprehend and accept the model of the physician-patient relationship in which the family members play an important role in informed consent. This implies that Chinese physicians may encounter ethical dilemmas when conducting informed consent. As mentioned above, in some cases, family members may request that physicians conceal the patient’s condition from the patient. Although doctors are allowed to inform family members rather than patients of their conditions in some special situations, [26, 27] there is no explicit provision of law that doctors can deliberately conceal the patient’s condition from the patient based on the request of the family members. Therefore, what should a doctor do when the family members’ demands conflict with the patient’s right to be fully informed? In addition, when a patient explicitly states that they do not want their family members to be involved, how should a doctor decide between the patient’s privacy and the family members’ requests for the patient’s information? And when a family member’s decision does not seem to fit the patient’s best interests, what should a doctor do? These ethical dilemmas and the challenges they cause for physicians will be the focus of our research.

As mentioned above, Chinese physicians are required to conduct informed consent to respect the patient’s autonomy. However, such requirements emerged gradually at the turn of the 21st century under relevant legal and ethical principles. At the legal level, the provision of informed consent in surgical procedures was first included in the Medical Practitioners Law of the People’s Republic of China in 1998 [28]. In terms of medical ethics education, mainstream textbooks gradually began to include informed consent as an ethical principle in the early 21st century. Medical ethics education is compulsory course in all medical universities in China. And the course is usually scheduled for undergraduate medical students in their junior or senior year. Thus, the moral distress felt by doctors who received medical education and entered clinical work after 2000, i.e., those in the under 35 age group, may be more pronounced than that felt by senior doctors. At the same time, these young doctors will become the backbone of the medical field in a decade or two, which means that their attitudes toward the “doctor-family-patient” relationship model will reflect the attitude of China’s medical community toward this model in the next few decades, as well as their responses to the corresponding ethical dilemmas.

Therefore, our study targets young doctors (under the age of 35) to investigate their attitudes and reactions to the above ethical dilemmas and the reasons behind their responses to them. This study is the first large-scale study of doctors’ attitudes and reactions to the “doctor-family-patient” model of the physician-patient relationship in China in the past decade, and the first to be conducted among young doctors under the age of 35.

China has multiple, distinct medical education pathways that can last from 5 to 11 years [29]. The normal age for the students who start a 5-year Bachelor of Medicine degree (MB) is 18. After MB program, they are qualified for the Medical Licensing Examination and further pursuing a 3-year Master of Medicine degree (MM). In fact, most medical students are able to obtain their medical license at the age of 23 before starting the MM program. The MM program usually include a 3-year general training which is required for physicians to be solely responsible for the patients. In our study, all participants have completed their MB program and hence, most of them have obtained the license. However, not all participants can independently manage patients considering that some of them are in their MM level. Even for these participants, they have played assistant role under supervision of high-hierarchy physicians in the physician-patient communication. Thus, all participants would have already experienced such model of physician-patient relationship.

## Methods

### Study design

This study was conducted from June 11, 2022, to September 20, 2022. Data were collected through an online survey using a snowball sampling method. The target population is doctors of all grades with clinical experience in various departments from 3 A hospitals, the highest normal level hospitals in the Chinese healthcare system.

### Questionnaires

The questionnaire was developed by consulting several literature including the theoretical discussions of the “doctor-family-patient” model of physician-patient relationship and some qualitative studies [5, 14, 19, 30]. In addition, a couple of young physicians were interviewed as the pre-study. The interview also contributed to the content of questionnaire.

The questionnaire consisted of 31 multiple-choice questions and took approximately 10 min to complete. The questions covered four major parts: the participants’ basic information, the fulfillment of the obligation to fully inform, who will be informed, ethical dilemmas in decision-making (Supplementary 1), and 10 questions related to the content of this article. The results of these 10 questions are included in this paper. Other questions will be presented and discussed in a subsequent article, *The requirements of fully informing and the reaction to patient’s decisions: a questionnaire study on Chinese doctors.*^1^

### Ethical considerations

Participants’ identifiable personal information was withheld from the questionnaire results data. The first page of the questionnaire stated the purpose of the study, its use, and the contact information of the person in charge. Informed consent was obtained from all participants (Supplementary 2). The study was also approved by the Ethics Committee of Nankai University (NKUIRB2022095).

### Data analysis

Data were imported into IBM SPSS version 25 for statistical analysis. Descriptive statistics were performed for each variable separately by the respondent. Cross tabulation and Pearson’s chi-squared test were used to analyze the differences between types of patients for categorical variables, and a *p*-value < 0.05 was considered statistically significant.

## Results

We obtained a total of 421 data sets, of which 368 met the age requirements for this study. The participants included 155 males (42.1%) and 213 females (57.9%). The minimum age was 21 years old, the maximum age was 35 years old, and the average age was 27.6 years old. In terms of education level, 118 participants (32.1%) held a bachelor’s degree, 217 participants (59%) held a master’s degree, and 33 participants (8.9%) held a doctoral degree. Finally, their professional titles were distributed as follows: 171 participants (46.5%) were resident physicians, 126 participants (34.2%) were attending physicians, 70 participants (19%) were associate chief physicians, and 1 participant (0.3%) was a chief physician (Table 1).

**Table 1:** Demographic information

Demographic	N(%)
Gender	
Male	155(42.1)
Female	213(57.9)
Age	
Min	21
Max	35
Average	27.6
	(95%CI:27.2–27.9)
Years of clinical experience	
Min	1
Max	10
Average	3.3
Educational level	
Bachelor’s degree	118(32.1)
Master’s degree	217(59.0)
Doctoral degree	33(8.9)
Position	
Interns	171(46.5)
Resident physician	126(34.2)
Attending physician	70(19.0)
Chief physician	1(0.3)
Total	368

**Fig. 1:**
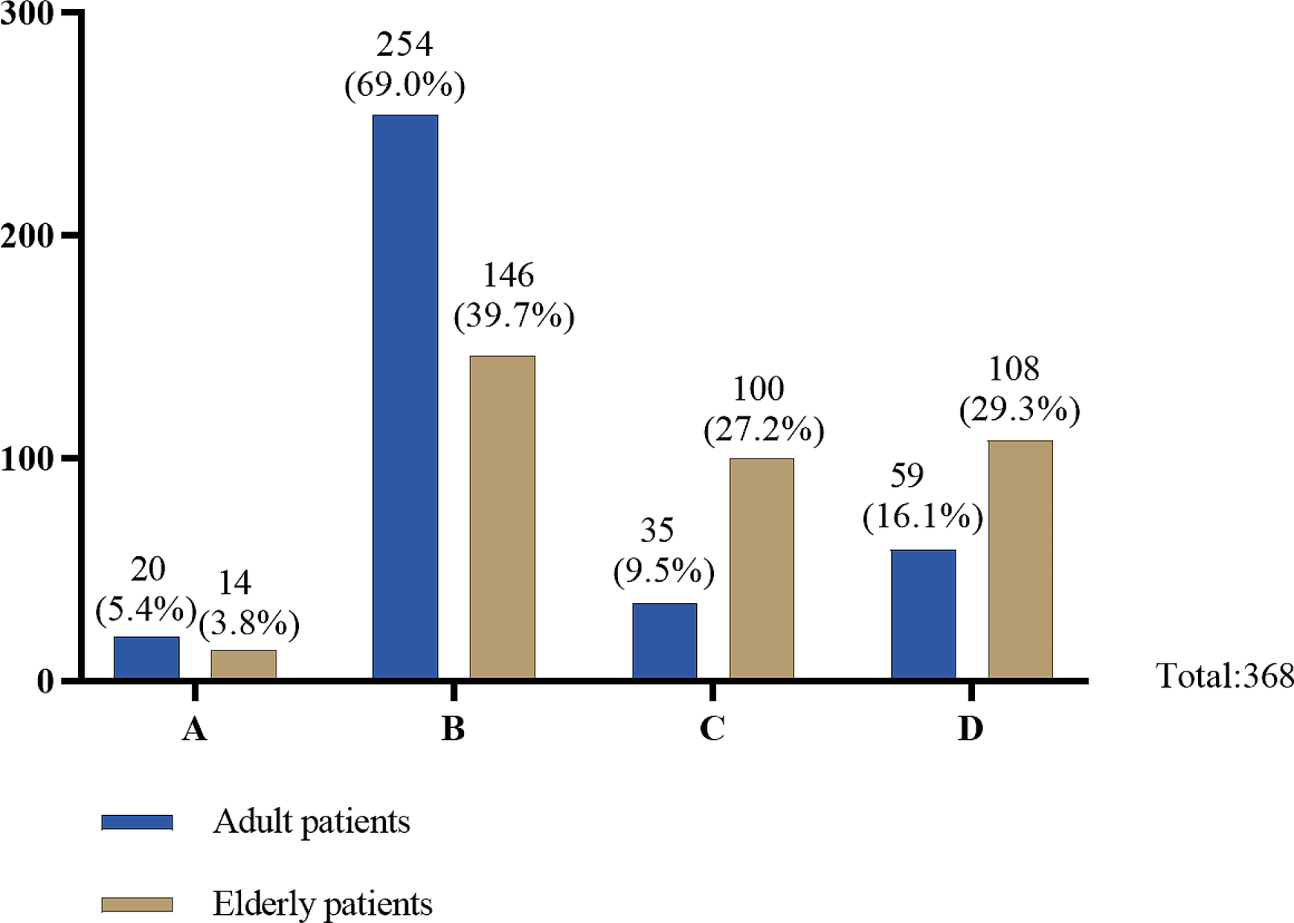
Different responses of doctors for different patients when informing patients who are accompanied by family members (or children) about a significant(severe) medical condition. (Table 2, Q1)

Our data (Table 2, Q1, Fig. 1) showed that only 20 doctors (5.40%) stated “informing the patient alone is sufficient” when it comes to informing adult patients of their serious conditions. 254 doctors (69.0%) stated that “unless the patient explicitly expresses a desire for their family members to remain uninformed, the family members will be informed”, while 35 doctors (9.5%) stated that “even if the patient explicitly expresses a desire for their family members to remain uninformed, the family members will be informed”. The remaining 59 doctors (16.1%) would “inform the family members first and let them inform the patient”. When facing elderly patients (60 and over [31]) with decision-making capacity, the situation was significantly different (Table 2, Q1, Fig. 1; Table 3). Only 14 doctors (3.8%) stated that “informing the patient alone is sufficient”. Of those surveyed, 146 doctors (39.7%) chose to “inform the patient’s children^2^ unless the patient explicitly expresses a desire for their children to remain uninformed”, while 100 doctors (27.2%) stated that “even if the patient explicitly expresses a desire for their family members to remain uninformed, the children will be informed”. Of respondents, 108 doctors (29.3%) would “inform the patient’s children first and let them inform the patient”. In general, many respondents who chose A (“inform the patient alone is sufficient”) or B (“inform the patient’s children unless the patient explicitly expresses a desire for their children to remain uninformed”) in the first question chose C (“even if the patient explicitly expresses a desire for their family members to remain uninformed, the children will be informed”) or D (“inform the patient’s children first and let them inform the patient”) in the second question (Table 3). By contrast, most respondents who chose C and D in the first question made the same selection (80% and 76.27%) in the second question (Table 3).

**Table 2:** Different responses of doctors when it comes to informed consent for different patients

Question	Options	Type of patients(N/%)		P
		Adult patients	Elderly patients *	
Q1:What will you do when informing patients who are accompanied by family members (or children)about a significant(severe) medical condition?	A.I will inform the patient himself/herself only	20(5.4)	14(3.8)	<0.001
	B. I will ensure the family is equally informed unless the patient explicitly expresses a desire for their family members to remain uninformed	254(69.0)	146(39.7)	
	C. I will ensure the family is equally informed even if the patient explicitly expresses a desire for their family members to remain uninformed	35(9.5)	100(27.2)	
	D. I will inform the family members first and let them inform the patient	59(16.1)	108(29.3)	
	Total	368	368	
Q2: What is the primary reason to ensure the family members are equally informed? (For those who did not select A in Q1)	A.A major medical condition can have an impact on the whole family, so families also have the right to know	144(41.4)	128(36.2)	
	B. Informing family members to let them to discuss with patients can help patients make better decisions	139(39.9)	180(50.8)	
	C. Such a strategy can help me to avoid medical disputes and preventing family members from holding doctors accountable on the grounds of not being informed	62(17.8)	44(12.4)	
	D. It is required by supervising physicians	1(0.3)	0	
	E. Other	2(0.6)	2(0.6)	
	Total	348	354	
Q3: What would you do if family members asked you to conceal the patient’s medical condition by claiming that it is in the patient’s best interest?	A. I will respect the views of the family and cooperate with them in concealing the condition from the patient	270(73.4)	293(79.6)	
	B. I will refuse it and let the family know that it violates professional ethics	73(19.8)	55(14.9)	
	C. Depends on the situation	21(5.7)	18(4.9)	
	D. Report to the supervisors and follow their instructions	4(1.1)	2(0.6)	
	Total	368	368	

* Except for elderly patients with Alzheimer’s disease and other conditions that affect their ability to make decisions

**Table 3:** Cross (Chi-square) analysis results of Table 2 Q1

Type of patients(N/%)	Options	Adult patients				Total	χ2	*p*
		A	B	C	D			
**Elderly patients**	A	6(30.00)	6(2.36)	0(0.00)	2(3.39)	14(3.80)	170.317	0.000**
	B	7(35.00)	131(51.57)	4(11.43)	4(6.78)	146(39.67)		
	C	1(5.00)	63(24.80)	28(80.00)	8(13.56)	100(27.17)		
	D	6(30.00)	54(21.26)	3(8.57)	45(76.27)	108(29.35)		
**Total**		20	254	35	59	368		

** *p* < 0.01

When asked about the primary reason for ensuring that family members are informed about the medical condition of the adult but not elderly patients (Table 2, Q2), 144 doctors (41.4%) believed that “major medical conditions can have an impact on the whole family, so families also have the right to know”, 139 doctors (39.9%) chose “informing family members to let them to discuss with patients can help patients make better decisions”, and 62 doctors (17.8%) chose “avoiding medical disputes and preventing family members from holding doctors accountable on the grounds of not being informed”. The proportion of doctors who cited the reasons for ensuring that family members are informed about the medical condition of elderly patients is slightly different (Table 2, Q2). Of those doctors, 180 (50.8%) believed that “informing adult children and involving them in medical decision-making can help patients to make better decisions”, 128 (36.2%) considered the overall impact on the patient’s family, and 44 (12.4%) chose “avoiding medical disputes and preventing family members from holding doctors accountable on the grounds of not being informed”.

We further analyzed the data on doctors who chose “avoiding medical disputes and preventing family members from holding doctors accountable on the grounds of not being informed” (Table 4). We found that compared to doctors who chose other options, a larger proportion (31.4%) of doctors who chose to inform family members even when adult but not elderly patients explicitly stated that they did not want their family members to be informed cited “avoiding medical disputes and preventing family members from holding doctors accountable on the grounds of not being informed”. However, there was no significant difference in this proportion when it came to elderly patients (13.0%).

**Table 4:** Percentage distribution of doctors considering exemptions primarily (Doctors who selected C in Q2)

		Total (N)		Considering exemptions primarily (N/%)	
Question	Options	Adult patients	Elderly patients *	Adult patients	Elderly patients *
What will you do when informing patients who are accompanied by family members (or children) about a significant(severe) medical condition?	A.I will inform the patient himself/herself only	20	14	Skip	Skip
	B. I will ensure the family is equally informed unless the patient explicitly expresses a desire for their family members to remain uninformed	254	146	42(16.5)	16(11.0)
	C. I will ensure the family is equally informed even if the patient explicitly expresses a desire for their family members to remain uninformed	35	100	11(31.4)	13(13.0)
	D. I will inform the family members first and let them inform the patient	59	108	9(15.3)	15(13.9)
**Total**		368	368	62	44

* Except for elderly patients with Alzheimer’s disease and other conditions that affect their ability to make decisions

**Fig. 2:**
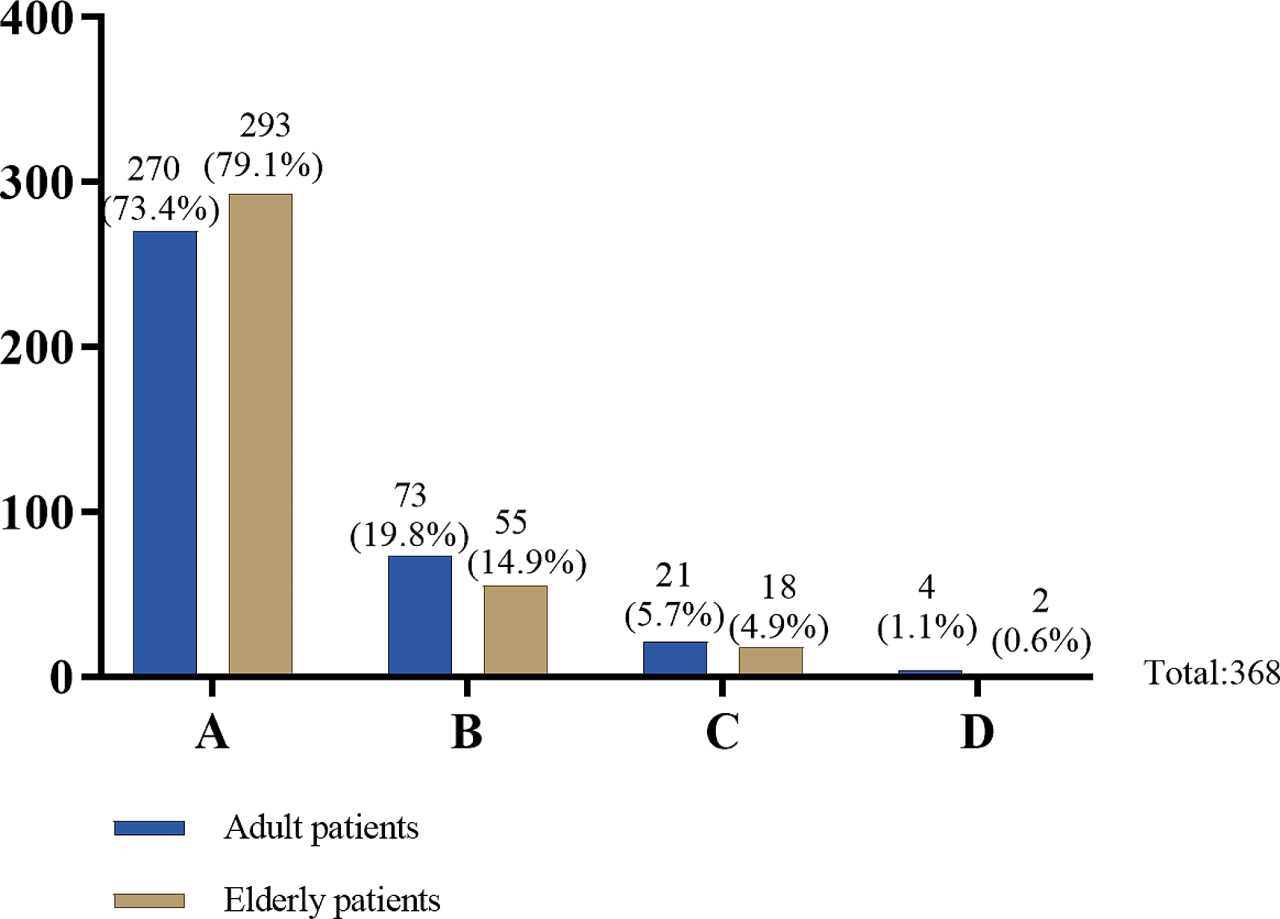
Different responses of doctors for different patients when family members asked doctors to conceal the patient’s medical condition by claiming that it is in the patient’s best interest. (Table 2, Q3)

When family members asked doctors to conceal the patient’s medical condition “for the best interests of patients”, 270 doctors (73.4%) chose to “respect the views of the family and cooperate with them in concealing the condition from the patient”, while 73 doctors (19.8%) explicitly refused the suggestion and advised the family that this violated professional ethics. In addition, 21 doctors (5.7%) tended to make situation-specific analyses, and 4 doctors (1.1%) would report to their superiors and follow their instructions (Table 2, Q3, Fig. 2). When faced with elderly patients who have decision-making capacity, the attitude of doctors did not significantly change. Of the respondents, 293 doctors (79.6%) chose to cooperate with adult children in concealing the patient’s medical condition, 55 doctors (14.9%) explicitly refused, and 18 doctors (4.9%) based the decision on the situation, while 2 doctors (0.6%) followed the instructions of their superiors (Table 2, Q3, Fig. 2).

## Discussion

Firstly, Chinese doctors pay extra attention to informing the patient’s family, which may not be in the patient’s best interests. In contrast to previous studies, our study reflects for the first time the balance that doctors consider between the patient’s right to know and their family members’ right to know. When faced with an adult but not elderly patient, most doctors (69%) would regard the family’s right to know as equally important to the patient’s right to know when informed of a severe medical condition. One-quarter of the respondents give extra weight to the family’s right to know, with some believing that it takes priority over the patient’s right to know (16%) and some even thinking that it is more important than the patient’s right to privacy (9.5%). Only 5.4% of respondents believe that it is only necessary to ensure the patient’s right to know.

It can be seen that Chinese doctors place a high priority on keeping patients’ families informed, but not exclusively for the best interests of the patient. According to the existing literature, patients, especially old patients, would not refuse their family members to involve in their medical decision making [5, 19, 32]. Three reasons were most mentioned: family-oriented tradition in China, the patient’s ability to understand the information, and the patient’s economic situation. Generally, both patients and their family members believe that this model in which the family have the patient’s information and take part in the patient’s medical decision-making fits the patient’s best interests. However, how physicians perceive this model is slightly covered by previous studies. Our study shows that only about 40% of participants’ primary reason for ensuring that family members are informed is for the interests of the patient; about 41% is for the consideration of the overall impact on the patient’s family; and about 18% is for self-protection considerations. Although the interests of the patient and the patient’s family are often consistent, factors other than the patient’s interests should not be the primary consideration for doctors as the requirement of medical professional ethics. As mentioned above, influenced by the family-oriented culture, family members play an important role in medical activities in China, which creates a so-called “doctor-family-patient” model of the physician-patient relationship. However, this can be morally accepted and defended because it is considered the way to maximize the patient’s interests in the Chinese cultural context. As doctors, the emphasis on the family’s right to know should be for promoting the patient’s best interests, rather than for weakening the consideration of the patient’s interests.

In addition, exemption considerations were particularly evident among respondents who felt that the family’s right to know was more important than the patient’s right to privacy. Our data suggest that the exclusion of liability has become an important reason for doctors to value family members’ right to information, which is contrary to their professional ethics. Although the subjective reasons for this phenomenon lie in the lack of professional ethics of physicians who have failed to consistently prioritize the patient’s interests, objective factors such as tense doctor-patient relationships cannot be ignored. Over the past decade, there have been unrelenting incidents of violence against doctors [33, 34]. The Lancet published two editorials in 2010 and 2020 calling for concern about the personal safety of Chinese doctors [35, 36]. Tensions in the doctor-patient relationship have led to a decline in trust between doctors and patients, and doctors have had to consider how to avoid getting themselves into potential disputes when facing patients and their families. This is particularly evident in extreme cases, such as when doctors delayed performing a cesarean section on a woman who died in labor, without obtaining her husband’s consent [37]. In the case, the pregnant woman was suggested by her physician to have a C- section immediately. However, her husband insisted a natural birth and refused to sign on the informed consent form. The physician didn’t perform a C-section on the woman as the family’s informed consent was the necessary for the operation. Then the pregnant woman died in labor. Fear of complaints from the patient’s husbands would be the main reason that the physician delayed the operation.

Secondly, our study reveals for the first time that Chinese doctors treat adult (but not elderly) patients and elderly patients differently when it comes to informing family members. Our data showed that compared to adult but not elderly patients, Chinese physicians tend to place greater emphasis on the family’s right to know (27.2% vs. 9.5%) with elderly patients, even if they have the capacity for decision-making, and consider it more beneficial to the patient (50.8% vs. 39.9%). The reasons for this difference in treatment mainly stem from two factors: the emphasis on filial obligation in traditional Chinese culture and the consideration of the education level of the elderly. First, influenced by traditional Confucian culture, Chinese society places great importance on the obligation of children to support and care for their parents [14]. Adult children accompanying their parents to medical appointments is often seen as a sign of a harmonious parent-child relationship and filial piety. Elderly patients are also often pleased to see their children show their concern by being informed and involved in decision-making. In such cases, doctors are more likely to be convinced that their children represent the best interests of their parents and are therefore more inclined to ensure that their children are informed in the interest of their elderly patients. In addition, the elderly population in China has a lower level of education. According to the 7th National Population Census of China in 2020, only 13.9% of the population aged 60 and above had a high school education or above [38]. The level of education significantly limits the elderly patient’s understanding of their medical condition, especially when medical terms are involved in the physician’s explanation [19]. In such cases, ensuring that family members are informed is more beneficial for the patient’s decision-making and subsequent treatment.

Thirdly, when family members request that doctors withhold information from patients “in the best interest of the patient”, the majority (over 70%) of participants choose to comply with the request, although this may cause them distress. The practice of benevolently withholding information from patients is not uncommon in medical history [39]. However, since the 1950s, with the shift in the doctor-patient relationship and the emphasis on patient autonomy, this practice has been criticized as paternalism and has been gradually replaced by informed consent [40, 41]. In current medical practice, the professional ethics of doctors in many countries explicitly prohibit doctors from withholding information from patients [42, 43]. In Western countries, on the patient’s family side, it is not common for them to request doctors to withhold information from patients. On the doctor’s side, it is difficult to imagine that doctors agree and comply with such requests. For example, Anne Lapine and her colleagues reported an interesting case in which the wife, daughter, and son-in-law of a Chinese patient requested that an American-born physician withhold information regarding a terminal diagnosis. The family felt that if the patient was told he had cancer, then his spirit would be broken. The Ethics Committee invited an American-born Chinese physician as a guest consultant. The consultant confirmed that it is common in Chinese culture for the family to request physicians to withhold a terminal diagnosis to protect the patient’s feelings [44].

However, even in China, complying with family members’ requests to withhold information from patients may cause distress to doctors. In our pre-study, the interviewees mentioned that. It is also showed in existing literature [45–47]. The family’s requests put the physician in a dilemma. On the one hand, withholding information from patients not only violates professional ethics but also violates relevant laws. The current professional ethics and laws all require doctors to fulfill their obligation to inform patients fully and respect patients’ autonomy [26, 27]. Although in some special cases doctors are allowed to inform family members rather than patients themselves, no law or ethical rule explicitly states that doctors can withhold information from patients based on family members’ requests [26, 27]. On the other hand, although most doctors acquiesce to the power of family members to make decisions for patients and indicate their willingness to comply with family members’ requests to withhold information from patients, it can be imagined that some of these doctors do so for the sake of exemption rather than the best interests of the patient. For doctors who consider the best interests of the patient, deciding whether to comply with family members’ requests to withhold information from patients is not an easy task. It requires the doctor to consider the patient’s condition, the patient’s mental capacity, the patient’s rapport with the family, and the impact of withholding information on subsequent treatment. This is probably why some respondents stated that it depends on the specific situation.

## Limitations

Our study has some limitations and deficiencies. First, our participants were all recruited from 3 A hospitals, which have a relatively high concentration of medical resources and a relatively high volume of patients. However, 3 A hospitals’ situation is limited to show the whole picture of the physician-patient relationship models considering the proportion of 3 A hospitals is low in China. Future studies should also focus on hospitals or medical institutions of lower levels. Differences in management systems, patient volume, and types of doctor-patient relationships between hospitals of different levels may lead to variations in results. Second, although snowball sampling was the most appropriate and efficient way to collect data under the covid-19 prevention and control requirements in China last year, it has its unavoidable disadvantages as a non-probability sampling method. Such as sample selection bias, limited generalizability, difficult to estimate sampling error and so on. Third, our data have been representative of young doctors as possible on the basis of publicly available data. However, our sample size was not large enough, and factors such as age, department, and region were not completely balanced, which may have resulted in some bias.

## Conclusions and practical implications

Our study is the first large-scale study of young doctors’ (under the age of 35) attitudes and reactions to the “doctor-family-patient” model of the physician-patient relationship in China. It shows the doctors’ balance weighing the patient’s right to know against the family’s members’ right to know, as well as the patient’s right to privacy against the family’s requests for the information. It also shows the doctor’s responses to the requests from family members to withhold information from patients and the reasons behind such responses. Our study reflects the potential moral distress caused by such a model. In terms of practical implications, it is necessary to increase clinical ethics training to promote doctors’ professionalism considering that some doctors are not motivated for the best interests of the patient. In addition, the ethical dilemmas faced by doctors cannot be ignored. Perhaps it could be helpful to have different rules for informed consent for different types of populations, i.e., adult and elderly patients. Furthermore, communication techniques such as SPIKES protocol [48] and CST [49] may be useful aids for doctors when communicating with patients and their families.

### Electronic supplementary material

Below is the link to the electronic supplementary material.

Supplementary Material 1

Supplementary Material 2

## Data Availability

The datasets used and/or analysed during the current study are available from the corresponding author on reasonable request.

## Footnotes

1 The article is being written.

2 Most of the time, it is adult children who play as family members to accompany their parents to visit hospitals.
