# Supplementary material for: Family roles in informed consent from the perspective of young Chinese doctors: a questionnaire study
Source: BMC Med Ethics. 2024 Jan 3;25:2. doi: 10.1186/s12910-023-00999-6 (PMC10765650; doi:10.1186/s12910-023-00999-6)
Supplement: Supplementary file 1 — Supplementary Material 1 [file 12910_2023_999_MOESM1_ESM.docx]

**QUESTIONNAIRE**

**1. Personal Information**

1.1 Your age ()

1.2 Your gender

A male ()

B. Female ()

1.3 The highest degree you have obtained

A. Undergraduate degree ()

B. Master's degree ()

C. Doctor degree ()

D. Others _______

1.4 Your Marital Status

A. Married ()

B. Unmarried ()

C. Divorced ()

1.5 Your current position/title

A. Graduate students in training ()

B. Residency ()

C. Attending physician ()

D. Chief physician ()

1.6 Your current department ()

1.7 Number of years you have worked in the clinic ()

1.8 The province where you work: _________

1.9 Have you received specific training related to "informing patients"?

A. None ()

B. There are ()

**2. The duty to disclose**

2.1 In clinical practice, physicians have a duty to provide adequate information to patients so that they can make rational decisions based on their medical condition and their situation. As a physician, are you able to provide adequate information to your patients?

A. Yes. I’m always able to do so ()

B. In most cases, I can fulfil the requirements of informed consent ()

C. In a few cases, I’m able to do so ()

D. No. I rarely provide adequate information to patients ()

2.2 What are the factors that influence you to fail to let patients be adequately informed? (Multiple choice)

A. The age of the patient ()

B. The complexity/severity of the patient’s condition ()

C. The patient’s extreme emotional responses and their poor coping skills ()

D. The patient’s educational background and family environment, etc. ()

E. Too busy for work to provide information in details ()

F. It is not necessary to explain fully considering that the patient cannot understand ()

G. Pressure from the high-level physicians or the limited medical resources ()

2.3 What is your initial purpose/objective for fulfilling the duty of providing adequate information in your clinical work?

A. To avoid medical disputes and skim off responsibility ()

B. To help patients better understand their condition for decision-making ()

C. It is required by the supervising physicians ()

D. Other _________ ()

2.4 What information do you think is important to inform patients? (Multiple choice)

A. The diagnosis, best treatment modality, risks, complications and sequelae, etc. ()

B. Other derived knowledge about the disease, such as the causes of its onset, principles of treatment ()

C. Alternative treatment modalities ()

D. The cost of treatment, rehabilitation and so on ()

2.5 As a physician, do you endorse the duty to provide adequate information to patients in the current healthcare environment?

A. Recognition. Patients should be fully informed. ()

B. Do not think so. Please fill in the reason __________. ()

**3. Whom to inform**

3.1 What will you do when informing adult (non-elderly) patients who are accompanied by family members about a significant(severe) medical condition?

A. I will inform the patient himself/herself only ()

B. I will ensure the family is equally informed unless the patient explicitly expresses a desire for their family members to remain uninformed ()

C. I will ensure the family is equally informed even if the patient explicitly expresses a desire for their family members to remain uninformed ()

D. I will inform the family members first and let them inform the patient ()

3.2 For adult (non-elderly) patients, what is the primary reason to ensure the family members are equally informed? (For those who did not select A in 3.1)

A. A major medical condition can have an impact on the whole family, so families also have the right to know ()

B. Informing family members to let them to discuss with patients can help patients make better decisions ()

C. Such a strategy can help me to avoid medical disputes and preventing family members from holding doctors accountable on the grounds of not being informed ()

D. It is required by supervising physicians ()

E. Other _________ ()

3.3 For adult (non-elderly) patients, what is your primary reason to inform the family members first and let them inform the patient? (For those who selected D in 3.1)

A. The family explicitly requested that ()

B. I’m worried that the patient may lose control of emotions and then have extreme behaviour considering that they may not accept the results ()

C. I’m worried that the family may accuse the doctor if the patient is out of control ()

D. Other _________ ()

3.4 For adult (non-elderly) patients, what would you do if family members asked you to conceal the patient’s medical condition by claiming that it is in the patient’s best interest?

A. I will respect the views of the family and cooperate with them in concealing the condition from the patient ()

B. I will refuse it and let the family know that it violates professional ethics ()

C. Other __________ ()

3.5 For elderly patients with adult children accompanying them (except for elderly patients with Alzheimer’s disease and other conditions that affect their ability to make decisions), what would you do when you planned to inform the patients?

A. I will inform the patient himself/herself only ()

B. I will ensure the children are equally informed unless the patient explicitly expresses a desire for their children to remain uninformed ()

C. I will ensure the children are equally informed even if the patient explicitly expresses a desire for their children to remain uninformed ()

D. I will inform the children first and let them inform the patient ()

3.6 For the above elderly patients, what is the primary reason to ensure their children are equally informed? (For those who did not choose A in 3.5)

A. Medical decision-making for elderly patients is often in the hands of their children, and informed children are necessary for follow-up treatment ()

B. Informing children and letting them discuss with patients can help patients make better decisions ()

C. Such a strategy can help me to avoid medical disputes and prevent children from holding me accountable on the grounds of not being informed ()

D. It is required by supervising physicians ()

E. Other _________ ()

3.7 For the above elderly patients, what is your primary reason to inform the children first and let them inform the patient? (For those who selected D in 3.5)

A. The children explicitly requested that ()

B. I’m worried that the patient may lose control of emotions and have extreme behaviour considering that they may not accept the results ()

C. I’m worried that the children may accuse the doctor if the patient is out of control ()

D. Other _________ ()

3.8 For the above elderly patients, what would you do if their children asked you to conceal the patient’s medical condition by claiming that it is in the patient’s best interest??

A. I will respect the children’s views and cooperate with them in concealing the condition from the patient ()

B. I will refuse it and let the children know that it violates professional ethics ()

C. Other __________ ()

3.9 What would you do when informing minor adolescent (16-18 years old) patients who are accompanied by a parent about a significant medical condition?

A. I will inform the patient himself/herself only ()

B. I will inform the patient and his or her parent simultaneously or sequentially ()

C. I will inform the parent first and let him or her inform the patient ()

D. Other _______ ()

3.10 What would you do if you encountered a minor adolescent (16-18 years old) patient who explicitly stated that he or she did not want their parents to know about their medical conditions?

A. I will respect the patient’s wishes and not inform the parents ()

B. I will inform the parents ()

C. Other _______()

3.11 Why did you choose to inform the parents even though the above minor adolescent patients clearly indicated that they did not want their parents to be informed? (For those who chose B in 3.10)

A. The parents can help patients to make more reasonable choices ()

B. Minor adolescent patients’ parents have the right to know ()

C. Such a strategy can help me to avoid medical disputes and prevent parents from holding me accountable on the grounds of not being informed ()

D. It is requested by the supervising physicians ()

E. Other ________ ()

**4. What is your attitude toward the patient’s decisions**

4.1 In clinical practice, have you ever encountered situations in which the patient’s decision-making was inconsistent or even conflicting with your professional judgment in term of the treatment plans?

A. I have never encountered such situations ()

B. I have occasionally encountered such situations ()

C. I have frequently encountered such situations ()

D. I always encountered such situations ()

4.2 In clinical practice, what would you do if a patient’s decision was inconsistent or even in conflict with your professional judgment in terms of the treatment plans?

A. I would respect the patient’s decision and move to the next session ()

B. I would remind the patient of the risk involved in the plan again and confirm whether the patient has understood his or her decision ()

C. I would do my best to persuade/guide the patient to choose the alternative that I prefer ()

D. Other _________ ()

4.3 What would you do if, as a breast surgeon, one of your patients was diagnosed with breast cancer and it indicates that mastectomy fits the patient more than breast-conserving treatment? The patient had agreed to have a mastectomy but said that she would discuss the decision with her husband. A few days later, the patient, accompanied by her husband, visited the doctor again and said that she ultimately chose breast-conserving surgery because her husband wanted to preserve her breast. Then, you would:

A. Respect the patient’s choice and go to the next stage (perform breast-conserving treatment) ()

B. repeatedly remind of the risk involved in breast-conserving treatment and confirm whether the patient and her husband have understood their decision ()

C. try to persuade the patient and her husband to choose the alternative ()

D. Other _________ ()

4.4 If you did not persuade the patient to choose a better treatment plan in your professional opinion, then what factors influenced you?

A. The patient’s autonomous decisions should not be interfered with ()

B. I’m worried that I may be accused if the recommended treatment plan causes problems in subsequent treatment even if it is not my mistake ()

C. I’m worried that I may be accused by the patients and their families once they are not satisfied with the recommended treatments’ effects ()

D. Other __________ ()

4.5 In clinical practice, what would you do when patients and their families disagree on the treatment plans and the reasons?

A. I will try to stay out of it and let the patient and the patient's family work it out on their own. My reason is _______ ()

B. I will try to help the patient to convince the family. My reason is _______ ()

C. I will try to help the family to convince the patient. My reason is ________ ()

D. I will help the party whose decision fits my professional judgment to convince the other party. ()

E. Other ___________ ()

4.6 In clinical practice, does it confuse you when a patient’s decision does not fit your professional judgment? Or in other words, whether it is when you think you should have persuaded the patient to choose a treatment plan that, in your opinion, was better, but did not do so because of external factors (e.g., fear of liability, patient backlash later, etc.)?

A. I have never been confused due to that ()

B. I was occasionally confused due to that ()

C. I was often confused due to that ()

D. I was always confused due to that ()
