# Supplementary material for: Family roles in informed consent from the perspective of young Chinese doctors: a questionnaire study
Source: BMC Med Ethics. 2024 Jan 3;25:2. doi: 10.1186/s12910-023-00999-6 (PMC10765650; doi:10.1186/s12910-023-00999-6)
Supplement: Supplementary file 2 — Supplementary Material 2 [file 12910_2023_999_MOESM2_ESM.docx]

**The statistical correlation between respondents' answers and their background about Table 2 Q1**

**1.For Adult patients**

| **Questions** | **Options** | **Age** | | **Gender** | | **Education level** | | | **Position** | | | |
| --- | --- | --- | --- | --- | --- | --- | --- | --- | --- | --- | --- | --- |
|  |  | **21-27** | **28-35** | **male** | **female** | **Bachelor's degree** | **Master's degree** | **Doctoral degree** | **Interns** | **Resident physician** | **Attending physician** | **Chief physician** |
| What will you do when informing patients who are accompanied by family members (or children)about a significant(severe) medical condition? | A.I will inform the patient himself/herself only | 10(4.61) | 10(6.62) | 10(6.45) | 10(4.69) | 9(7.63) | 8(3.69) | 3(9.09) | 10(5.85) | 3(2.38) | 6(8.57) | 1(100.00) |
|  | B. I will ensure the family is equally informed unless the patient explicitly expresses a desire for their family members to remain uninformed | 157(72.35) | 97(64.24) | 97(62.58) | 157(73.71) | 81(68.64) | 151(69.59) | 22(66.67) | 120(70.18) | 90(71.43) | 44(62.86) | 0(0.00) |
|  | C. I will ensure the family is equally informed even if the patient explicitly expresses a desire for their family members to remain uninformed | 14(6.45) | 21(13.91) | 19(12.26) | 16(7.51) | 5(4.24) | 26(11.98) | 4(12.12) | 12(7.02) | 14(11.11) | 9(12.86) | 0(0.00) |
|  | D. I will inform the family members first and let them inform the patient | 36(16.59) | 23(15.23) | 29(18.71) | 30(14.08) | 23(19.49) | 32(14.75) | 4(12.12) | 29(16.96) | 19(15.08) | 11(15.71) | 0(0.00) |
|  | Total | 217 | 151 | 155 | 213 | 118 | 217 | 33 | 171 | 126 | 70 | 1 |
|  | χ^2^ | 6.820 | | 5.441 | | 9.613 | | | 23.856 | | | |
|  | *p* | 0.078 | | 0.142 | | 0.142 | | | 0.005 | | | |

**2. For Elderly patients**

| **Questions** | **Options** | **Age** | | **Gender** | | **Education level** | | | **Position** | | | |
| --- | --- | --- | --- | --- | --- | --- | --- | --- | --- | --- | --- | --- |
|  |  | **21-27** | **28-35** | **male** | **female** | **Bachelor's degree** | **Master's degree** | **Doctoral degree** | **Interns** | **Resident physician** | **Attending physician** | **Chief physician** |
| What will you do when informing patients who are accompanied by family members (or children)about a significant(severe) medical condition? | A.I will inform the patient himself/herself only | 11(5.07) | 3(1.99) | 11(7.10) | 3(1.41) | 7(5.93) | 6(2.76) | 1(3.03) | 8(4.68) | 4(3.17) | 2(2.86) | 0(0.00) |
|  | B. I will ensure the family is equally informed unless the patient explicitly expresses a desire for their family members to remain uninformed | 92(42.40) | 54(35.76) | 54(34.84) | 92(43.19) | 43(36.44) | 89(41.01) | 14(42.42) | 66(38.60) | 55(43.65) | 25(35.71) | 0(0.00) |
|  | C. I will ensure the family is equally informed even if the patient explicitly expresses a desire for their family members to remain uninformed | 49(22.58) | 51(33.77) | 41(26.45) | 59(27.70) | 30(25.42) | 63(29.03) | 7(21.21) | 42(24.56) | 37(29.37) | 21(30.00) | 0(0.00) |
|  | D. I will inform the family members first and let them inform the patient | 65(29.95) | 43(28.48) | 49(31.61) | 59(27.70) | 38(32.20) | 59(27.19) | 11(33.33) | 55(32.16) | 30(23.81) | 22(31.43) | 1(100.00) |
|  | Total | 217 | 151 | 155 | 213 | 118 | 217 | 33 | 171 | 126 | 70 | 1 |
|  | χ2 | 7.384 | | 9.728 | | 4.236 | | | 6.616 | | | |
|  | P | 0.061 | | 0.021 | | 0.645 | | | 0.677 | | | |

**The statistical correlation between respondents' answers and their background about Table 2 Q3**

**1.For Adult patients**

| **Questions** | **Options** | **Age** | | **Gender** | | **Education level** | | | **Position** | | | |
| --- | --- | --- | --- | --- | --- | --- | --- | --- | --- | --- | --- | --- |
|  |  | **21-27** | **28-35** | **male** | **female** | **Bachelor's degree** | **Master's degree** | **Doctoral degree** | **Interns** | **Resident physician** | **Attending physician** | **Chief physician** |
| What would you do if family members asked you to conceal the patient’s medical condition by claiming that it is in the patient’s best interest? | A. I will respect the views of the family and cooperate with them in concealing the condition from the patient | 160(73.73) | 110(72.85) | 121(78.06) | 149(69.95) | 96(81.36) | 152(70.05) | 22(66.67) | 126(73.68) | 93(73.81) | 50(71.43) | 1(100.00) |
|  | B. I will refuse it and let the family know that it violates professional ethics | 43(19.82) | 30(19.87) | 28(18.06) | 45(21.13) | 14(11.86) | 51(23.50) | 8(24.24) | 36(21.05) | 24(19.05) | 13(18.57) | 0(0.00) |
|  | C. Depends on the situation | 12(5.53) | 9(5.96) | 6(3.87) | 15(7.04) | 8(6.78) | 10(4.61) | 3(9.09) | 8(4.68) | 8(6.35) | 5(7.14) | 0(0.00) |
|  | D. Report to the supervisors and follow their instructions | 2(0.92) | 2(1.32) | 0(0.00) | 4(1.88) | 0(0.00) | 4(1.84) | 0(0.00) | 1(0.58) | 1(0.79) | 2(2.86) | 0(0.00) |
|  | Total | 217 | 151 | 155 | 213 | 118 | 217 | 33 | 171 | 126 | 70 | 1 |
|  | χ^2^ | 0.171 | | 5.721 | | 11.271 | | | 3.804 | | | |
|  | *p* | 0.982 | | 0.126 | | 0.080 | | | 0.924 | | | |

**2.For Elderly patients**

| **Questions** | **Options** | **Age** | | **Gender** | | **Education level** | | | **Position** | | | |
| --- | --- | --- | --- | --- | --- | --- | --- | --- | --- | --- | --- | --- |
|  |  | **21-27** | **28-35** | **male** | **female** | **Bachelor's degree** | **Master's degree** | **Doctoral degree** | **Interns** | **Resident physician** | **Attending physician** | **Chief physician** |
| What would you do if family members asked you to conceal the patient’s medical condition by claiming that it is in the patient’s best interest? | A. I will respect the views of the family and cooperate with them in concealing the condition from the patient | 173(79.72) | 120(79.47) | 128(82.58) | 165(77.46) | 99(83.90) | 173(79.72) | 21(63.64) | 136(79.53) | 101(80.16) | 55(78.57) | 1(100.00) |
|  | B. I will refuse it and let the family know that it violates professional ethics | 33(15.21) | 22(14.57) | 21(13.55) | 34(15.96) | 12(10.17) | 34(15.67) | 9(27.27) | 28(16.37) | 16(12.70) | 11(15.71) | 0(0.00) |
|  | C. Depends on the situation | 9(4.15) | 9(5.96) | 6(3.87) | 12(5.63) | 7(5.93) | 8(3.69) | 3(9.09) | 6(3.51) | 8(6.35) | 4(5.71) | 0(0.00) |
|  | D. Report to the supervisors and follow their instructions | 2(0.92) | 0(0.00) | 0(0.00) | 2(0.94) | 0(0.00) | 2(0.92) | 0(0.00) | 1(0.58) | 1(0.79) | 0(0.00) | 0(0.00) |
|  | Total | 217 | 151 | 155 | 213 | 118 | 217 | 33 | 171 | 126 | 70 | 1 |
|  | χ^2^ | 2.015 | | 2.670 | | 10.049 | | | 2.801 | | | |
|  | *p* | 0.569 | | 0.445 | | 0.123 | | | 0.972 | | | |
